# Insights into the Periplasmic Proteins of *Acinetobacter baumannii* AB5075 and the Impact of Imipenem Exposure: A Proteomic Approach

**DOI:** 10.3390/ijms20143451

**Published:** 2019-07-13

**Authors:** Daniela Scribano, Valeria Marzano, Stefano Levi Mortera, Meysam Sarshar, Pamela Vernocchi, Carlo Zagaglia, Lorenza Putignani, Anna Teresa Palamara, Cecilia Ambrosi

**Affiliations:** 1 Department of Public Health and Infectious Diseases, Sapienza University of Rome, 00185 Rome, Italy; 2 Dani Di Giò Foundation–Onlus, 00193 Rome, Italy; 3 Unit of Human Microbiome, Bambino Gesù Children’s Hospital, IRCCS, 00146 Rome, Italy; 4 Department of Public Health and Infectious Diseases, Sapienza University of Rome, Laboratory Affiliated to Institute Pasteur Italia, Cenci-Bolognetti Foundation, 00185 Rome, Italy; 5 Microbiology Research Center (MRC), Pasteur Institute of Iran, Pasteur Ave 69, 1316943551 Tehran, Iran; 6 Units of Parasitology and Human Microbiome, Bambino Gesù Children’s Hospital, IRCCS, 00146 Rome, Italy; 7 San Raffaele Pisana, IRCCS, Telematic University, 00163 Rome, Italy

**Keywords:** *Acinetobacter baumannii*, carbapenem-resistance, β-lactamases, periplasmic proteome, oxidative stress, imipenem

## Abstract

Carbapenem-resistant *Acinetobacter baumannii* strains cause life-threatening infections due to the lack of therapeutic options. Although the main mechanisms underlying antibiotic-resistance have been extensively studied, the general response to maintain bacterial viability under antibiotic exposure deserves to be fully investigated. Since the periplasmic space contains several proteins with crucial cellular functions, besides carbapenemases, we decided to study the periplasmic proteome of the multidrug-resistant (MDR) *A. baumannii* AB5075 strain, grown in the absence and presence of imipenem (IMP). Through the proteomic approach, 65 unique periplasmic proteins common in both growth conditions were identified: eight proteins involved in protein fate, response to oxidative stress, energy metabolism, antibiotic-resistance, were differentially expressed. Among them, ABUW_1746 and ABUW_2363 gene products presented the tetratricopeptide repeat motif, mediating protein-protein interactions. The expression switch of these proteins might determine specific protein interactions to better adapt to changing environmental conditions. ABUW_2868, encoding a heat shock protein likely involved in protection against oxidative stress, was upregulated in IMP-exposed bacteria. Accordingly, the addition of periplasmic proteins from *A. baumannii* cultured with IMP increased bacterial viability in an antioxidant activity assay. Overall, this study provides the first insights about the composition of the periplasmic proteins of a MDR *A. baumannii* strain, its biological response to IMP and suggests possible new targets to develop alternative antibiotic drugs.

## 1. Introduction

*Acinetobacter baumannii* is a non-fermentative Gram-negative coccobacillus, commonly found in water, soil and normal flora of humans [1,2]. Initially considered a low virulent microorganism, the *A. baumannii* group has become one of the most concerning opportunistic pathogens in health-care settings worldwide, accounting for approximately 90–95% of clinical infections and nosocomial outbreaks (www.who.int; www.ecdc.europa.eu). Most *A. baumannii* infections include pneumonia, predominantly ventilator-associated, bacteremia, trauma and wound infections, urinary tract infections, meningitis and endocarditis, occurring mainly in immunocompromised patients admitted to intensive care units [1]. The exceptional advantage of *A. baumannii* over other nosocomial microorganisms is its ability to persist on medical device and equipment surfaces and rapidly develop resistance to most or all available antibiotics [1,2]. Although the carbapenem class of antibiotics was the optimal option for efficaciously treating *A. baumannii* infections [3,4], regretfully, rates of resistance to carbapenems are progressively increasing among *A. baumannii* clinical isolates [1]. The resistance to carbapenems is often conferred by a decreased influx and/or increased antibiotic efflux. The decreased outer membrane (OM) permeability is achieved by under-expression of porins involved in the antibiotic influx and increased drug efflux by efflux pumps [2]. Among others, the efflux pump mainly associated with carbapenem efflux is the three-component AdeABC pump (for *Acinetobacter* drug efflux), belonging to the resistance-nodulation-division (RND) superfamily [5]. Furthermore, a crucial role in carbapenem-resistance *A. baumannii* is given by carbapenem-hydrolyzing enzymes or carbapenemases due to their specific hydrolyzing activity against carbapenems [2,3]. The different classes of carbapenemases are present in *A. baumannii* around the world including class B, a subgroup of class D β-lactamases (carbapenem-hydrolyzing class D β-lactamases, CHDLs) as well as some β-lactamases from class A (i.e., GES, KPC) [2,3]. In multidrug-resistant (MDR) *A. baumannii* strains, β-lactamases reside within the periplasmic space [6].

The periplasm is a multipurpose compartment that includes a variety of enzymes and functions that carry on protein oxidation, folding and quality control [7]. The proteins residing within the periplasmic space are exported across the inner membrane mainly by the Sec or, to a lesser extent, by the twin-arginine translocation (TAT) pathways [8]. Specific Sec- and TAT-dependent signal peptides target unfolded or folded proteins to the specific translocation machinery, respectively [8]. In addition, some proteins lacking any apparent targeting signals are translocated across the inner membrane by the non-classical secretion pathway [9]. It is reasonable to hypothesize that, under the antibiotic treatment, the protein content of periplasmic space could be profoundly affected through the secretion and accumulation of different proteins involved in maintaining efficient cell functions.

Currently, proteomic studies on the cell envelope of MDR *A. baumannii* have been aimed at characterizing the outer and inner membrane sub-proteomes as well as the extracellular proteome under different growth conditions [10,11,12,13]. Instead, the composition and dynamics of *A. baumannii* periplasm remain largely uncharacterized. Therefore, the aim of this study was to get insights in *A. baumannii* periplasmic proteome and apply comparative proteomics to investigate the regulation of protein expression under sub-inhibitory concentration of imipenem (IMP). The fully sequenced MDR *A. baumannii* AB5075 strain, belonging to the ICL 1, was chosen for its antibiotic resistance profile and increased virulence in animal models [14,15]. Herein, we characterized, for the first time, the periplasmic proteins of strain AB5075 using a proteome approach. By comparing the periplasmic proteomes in the absence and presence of a sub-inhibitory IMP concentration, eight differentially expressed proteins involved in protein fate, responses to oxidative stress and energy metabolism were identified.

## 2. Results and Discussion

### 2.1. Assessment of Sub-Inhibitory Concentrations of IMP to Achieve Growth Curves Comparable to the Unexposed Control

**Figure 1:**
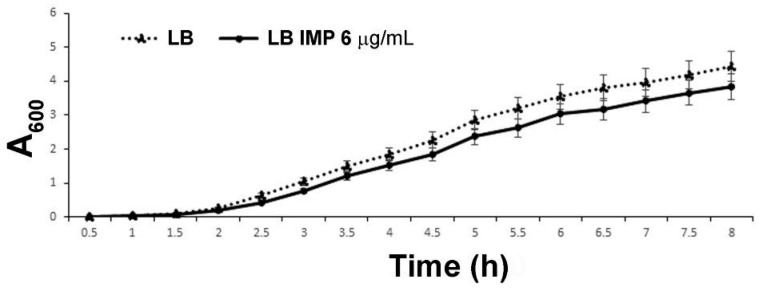
The growth curve of strain AB5075 cultivated in LB and LB supplemented with 6 µg/mL of IMP. The data are the means ± standard deviation from at least three independent experiments.

The clinical isolate strain AB5075 was selected as a model strain to study the periplasmic proteome as this multidrug resistant strain is known to be IMP resistant [14,16]. The full antibiotic-resistant profile is shown in Supplementary Material (Table S1). The growth curves of strain AB5075 grown in Luria-Bertani (LB) broth in the presence of 8, 6, 4, 2, 0 µg/mL of IMP were determined, measuring optical density (OD_600_) over a period of 8 hrs. The viable count and bacterial morphology were also monitored at the endpoint. IMP at a final concentration of 6 µg/ml allowed comparable growth curves with no statistical significance with the LB control (Figure 1), in accordance with the antibiogram value (Table S1). Moreover, no differences in bacterial viable count or morphology were detected between strain AB5075 grown in LB and in LB supplemented with IMP. Therefore, 6 µg/mL of IMP was used throughout the study.

### 2.2. Assessment of the Most Effective Method for Periplasmic Protein Extraction from A. baumannii Cells

**Figure 2:**
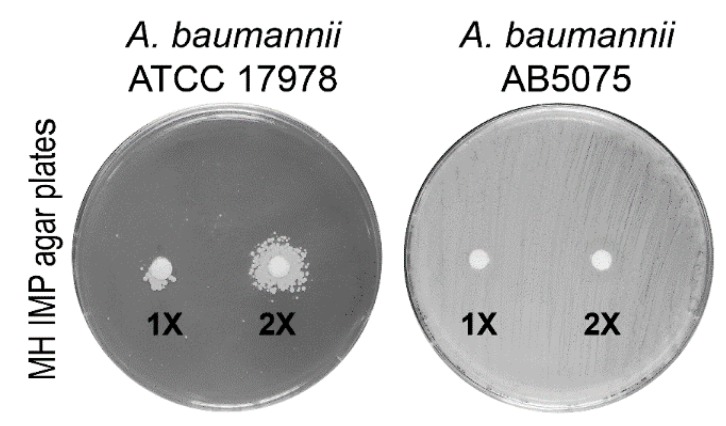
Microbiological plate assay showing the growth of the imipenem (IMP)-susceptible *A. baumannii* ATCC 17978 strain in the nearby of a disk soaked with periplasmic proteins from strain AB5075. The microbiological disks applied on the surface of the seeded plates contained 15 µL of 1× (20 µg/mL) or 2× (40 µg/mL) periplasmic fractions extracted using the sucrose and lysozyme method. The plates seeded with strain AB5075 served as the positive control, whereas disks containing 15 µL of the buffers used to extract periplasmic proteins were used as the negative controls (not shown).

The different cell-fractionation protocols have been developed to enrich the periplasmic protein pool in bacteria [17,18,19]. To establish the most efficient and comprehensive method to extract periplasmic proteins, three experimental approaches for periplasmic protein recovery were compared. The periplasmic enrichment was analyzed by a western blot using an antiserum against SurA from *Escherichia coli*. Unfortunately, the anti-SurA antiserum had no cross-reactivity with the *A. baumannii* SurA protein, likely reflecting the low percentage of homology between orthologous proteins (SurA*_E. coli_* vs. SurA*_A. baumannii_*, amino acid identity 118/428 (28%). Therefore, a plate assay to test which method of extraction ensured the recovery of the highest carbapenemase activity in the periplasmic fractions was set up (see Materials and Methods for details). Disks soaked with the three different periplasmic preparations were placed on the surface of IMP agar plates to allow the growth of the carbapenem-susceptible *A. baumannii* strain ATCC 17978 (Figure 2). The spheroplasting method based on sucrose and lysozyme showed the highest recovery of carbapenemases [19]. To assess the level of cytosolic, the inner or outer membrane contaminants, periplasmic proteins extracted with the sucrose and lysozyme method from strain AB5075 grown in LB, were analyzed both by gel-free and gel-based bottom-up proteomic strategies [20]. The amount of lysozyme used in the periplasmic fraction isolation step hampered the gel-free approach, as ion suppression occurrence during nano-High-Performance Liquid Chromatography-ElectroSpray Ionization-tandem mass spectrometry (nano-HPLC-ESI-MS/MS) analysis led to a reduced number of identified proteins. Thus, the gel-based proteomic strategy was chosen. Eleven bands from top to bottom of the Coomassie blue stained gel were cut. The proteins in each band were in-gel digested and obtained peptides were analyzed by nano-HPLC-ESI-MS/MS. From the 282 identified unique proteins, those experimentally characterized and established to be periplasmic and predicted by the presence of putative classical/non-classical signal peptides, were included and accounted for 39.8% (Table S2). The proteins with predicted transmembrane helices different from the short transmembrane helix in the N-terminus were excluded. These enrichment levels of periplasmic proteins are in line or above previous publications [21,22,23,24]. Undoubtedly, some extent of cell lysis occurred during preparation of periplasmic protein fractions, in that known cytoplasmic proteins were recovered, accounting for the 54.3% (Table S2). Conversely, the contamination of membrane proteins was low, accounting for 5.6%, (Table S2). It has been reported that periplasmic proteins accounted for 6% in fast-growing *E. coli* cells [25]. As the sucrose and lysozyme method allowed to achieve an enrichment in periplasm proteins of 39.8%, this extraction method was considered suitable and, therefore, was used throughout the study.

### 2.3. General Features and Differentially Expressed Periplasmic Proteins of A. baumannii AB5075 Grown in the Presence or Absence of IMP

**Figure 3:**
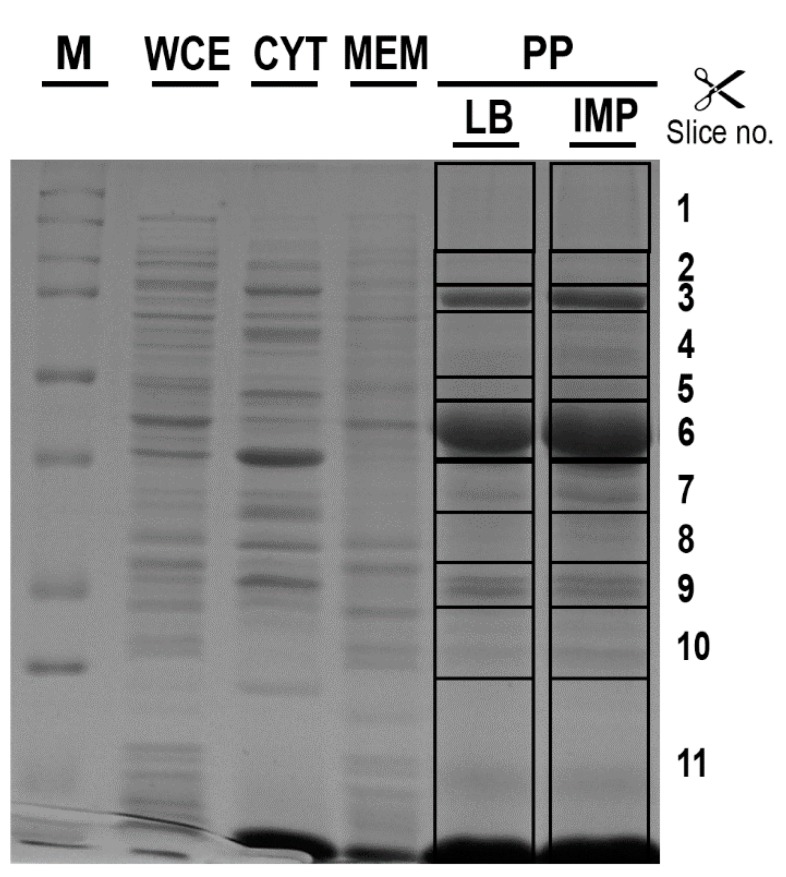
The different protein profiles of cell-fractionation from *A. baumannii* AB5075. The periplasmic enriched fractions (PP) extracted from strain AB5075 cultured in control (LB) or LB supplemented with 6 μg/mL IMP (IMP) were resolved by SDS-PAGE and stained with Coomassie blue R-250. The protein profiles from the whole cell extract (WCE), the soluble (CYT) and membrane-associated (MEM) fractions extracted from strain AB5075 cultured in LB supplemented with IMP are also shown for comparison. M, molecular mass standards in kDa are given on the left. The gel slices excised and subjected to in-gel digestion are indicated on the right side. The reported image is representative of three independent experiments.

**Figure 4:**
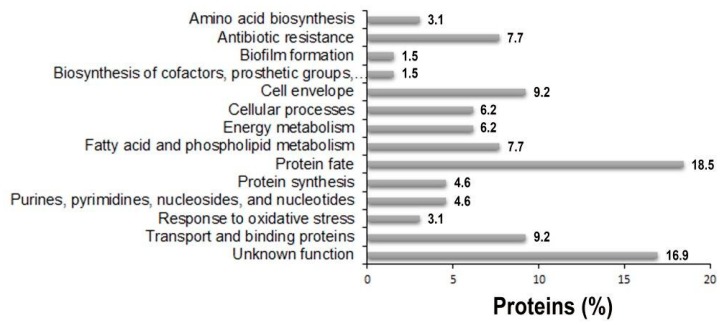
The distribution of functional categories of putative periplasmic proteins identified in the periplasmic fractions of strain AB5075 grown in the presence and the absence of IMP. The assignment to each functional category was based on TIGR annotation [http://www.tigr.org] and on available literature of single proteins.

To investigate periplasmic proteins differentially regulated in response to IMP exposure, periplasmic proteomes derived from strain AB5075 grown in LB and in the presence of sub-inhibitory concentration of IMP were compared using exponentially modified protein abundance index (emPAI) values [26]. Three independent periplasmic preparations were resolved on a mono-dimensional Sodium Dodecyl sulphate-polyacrylamide gel electrophoresis (SDS-PAGE) and stained with Coomassie blue (Figure 3). The gel-based proteomic analysis identified, on average, 232 unique proteins in LB and 288 in LB supplemented with IMP (data not shown). A range from 38.1 to 42.8% were proteins established or predicted to reside within the periplasmic compartment. Among those, 65 proteins were identified and found to be common in LB and LB supplemented with IMP (Table 1). Most of them (86.1%) possessed export signals of the Sec-type. Further, three proteins predicted to be Sec-dependent were also positive for a potential Tat signal peptide, without a detectable Tat motif (Table 1). From the remaining 8 proteins, 12.0% were considered periplasmic according to the SecretomeP server that predicts non-classical or leaderless secreted proteins which includes also lipoproteins [27]. Finally, the aconitase hydratase B was included in the periplasmic proteins on the basis of a previous publication [11]. The identified 65 periplasmic proteins were further analyzed and grouped according to functional categories (Figure 4 and Table 1). The 64.7% of the periplasmic proteins belonged to protein fate, transport and binding proteins, the cell envelope, fatty acid and phospholipid metabolism, antibiotic-resistance, energy metabolism and cellular processes categories (Figure 4 and Table 1). Conversely, those with an unknown function accounted for the 16.9% (Figure 4 and Table 1). Interestingly, among the 65 identified periplasmic proteins, 11 are encoded by genes essential for the growth on nutrient-rich medium, as LB, in strain AB5075, 2 non-essential and 1 gene previously annotated as a hypothetical protein that has no orthologue in strain ATCC 17978 [15] (Table 1). Our results provide evidence that this latter gene definitely expresses a protein although its function is still unknown.

#### 2.3.1. Antibiotic Resistance

**Figure 5:**
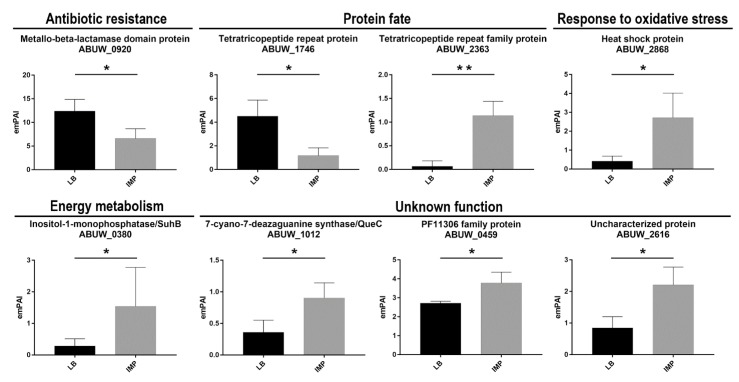
Semi-quantitative analysis of differentially expressed periplasmic proteins from strain AB5075 grown in LB or LB supplemented with IMP. The data are the means ± standard deviation from three independent experiments. The functional categories of periplasmic proteins identified assigned as previously described are shown. The emPAI and statistical significance values are shown. Asterisks represent p values evaluated by *t* test; ** *p* < 0.01, * *p* < 0.05.

Four proteins involved in antibiotic-resistance were identified, the cephalosporinase AmpC, the carbapenemases OXA-23 and GES-11 as well as the RND-type efflux pump AdeT (Table 1). No significant differences were observed in the expression of these proteins in the presence or absence of IMP. In the majority of *A. baumannii* clinical strains, the expression of β-lactamases is upregulated by upstream promoters located on insertion sequences, such as IS*Aba1* or IS*Aba125* [28,29]. In strain AB5075, an IS*AbaI* is present upstream the *blaOXA-23_,_* whereas *blaGES-11* is located in pAB50751 within a resistance island which also includes aminoglycoside, chloramphenicol and trimethoprim resistance genes [15]. Conversely, no insertion sequence was found upstream the AB5075 *ampC*, thereby leading to six times lower expression in comparison to those expressed from an upstream IS [15,28]. Nevertheless, a study demonstrated a variable level of AmpC, OXA-51-like carbapenemase and efflux pumps expression in IMP-induced *A. baumannii* mutants [30]. In strain AB5075, the deregulated co-expression of OXA-23 and GES-11 suggests that those enzymes are responsible for IMP resistance as well as the increased resistance to many other classes of antibiotics. The protein AdeT was previously shown to be involved in aminoglycoside resistance [31]. A slight increase in AdeT expression, although with no statistical significance was observed, suggests that its expression may contribute to IMP resistance. In the genomes of *A. baumannii* strains ATCC 17978 and AC0037, two *adeT* genes were found [32,33]. A search in the AB5075 genome revealed three additional genes encoding putative AdeT proteins, ABUW_0008, ABUW_0010 and ABUW_0394, sharing amino acid identity ranging from 62 to 32% with the AdeT herein reported. This high *adeT*-like gene content could indicate a wide protective ability of strain AB5075 from harmful substances, such as antibiotics and disinfectants. A fifth uncharacterized protein was identified, ABUW_0920 gene product, encoding a metal-dependent hydrolase of the β-lactamase superfamily (Table 1, Figure 5). The enzymes belonging to this superfamily include several hydrolytic enzymes that carry out a variety activities, including class B β-lactamases, hydrolases, lactonases, and persulfide dioxygenases (https://www.ncbi.nlm.nih.gov/Structure/cdd/cddsrv.cgi?uid = cd07726). Interestingly, the expression of ABUW_0920 was found to be significantly higher in the absence of IMP, thereby suggesting an enzymatic activity different from the hydrolysis of β-lactams (Table 1, Figure 5).

#### 2.3.2. Protein Fate

The disulfide isomerase DsbA and peptidyl-prolyl isomerases (PPIases) SurA and FklB were identified (Table 1). DsbA belongs to the Dsb system and catalyzes S-S bond formation on dozens of proteins containing S-S bonds due to its oxidizing redox potential and dithiol oxidase activity. In *E. coli*, substrates for DsbA are outer membrane proteins (OMPs), periplasmic proteins, β-lactamases, heat-stable enterotoxins, pilin proteins, and components of various secretion apparatuses involved in virulence [34,35]. For this reason, inactivation of the *dsbA* locus dramatically affects bacterial virulence in several pathogens [36,37,38,39]. In addition, DsbA participates in the protective response to oxidative stress, acting on misfolded proteins [35]. PPIases maintain substrates in a folding-competent state and catalyze the *cis-trans* isomerization of proline peptide bonds, thereby assisting protein folding. A range of periplasmic chaperone-proteases, such as MucD (DegP), PtrA, Prc, PrlC and PepN were also identified (Table 1). As reported for other bacteria, several chaperones, folding catalysts and proteases, are present with overlapping activities to control protein folding and protein quality in this cellular compartment. Further, the periplasmic lipoprotein-specific chaperone LolA was identified and together with SurA and MucD, was implicated in the correct assembly and incorporation of lipoproteins and OMPs, as reported for other Gram-negative bacteria [21,22,40,41]. Therefore, it is likely that the lack of significant variations between the two growth conditions is due to the redundancy of these chaperones. Conversely, we found two differently expressed proteins (encoded by ABUW-2363 and YbgF) possessing the tetratricopeptide repeat (TPR) structural motif [42], but sharing no amino acid sequence identity. YbgF was significantly more expressed in the absence of IMP, whereas ABUW_2363 in LB supplemented with IMP (Figure 5). Although ABUW_2363 has numerous specific orthologs in several species, regretfully, no function was inferred for these proteins. Instead, YbgF has a 100% identity with the Tol-Pal system protein YbgF from *Klebsiella pneumoniae* (UniprotKB entry A0A331U8H4). The lower percentage of identity (38–28%) was found with the BamD factor, a component of the BAM complex, from several species of proteobacteria, that have played a critical role in the BamA-mediated OMP folding pathway [43]. The genetic environment of the *ybgF* gene in strain AB5075 is different from its ortologous in *K. pneumoniae*, being unlinked to the *tol-pal* system but instead close to *recX*, *recA* and *bamA* genes. Therefore, it is tempting to speculate that bacteria maximizes biogenesis and assembly of OMPs under rich medium (LB) growth conditions, whereas downregulating them to decrease cell permeability during antibiotic stress exposure. A third protein containing a TRP motif, encoded by ABUW_0664, with no amino acid sequence identity with YbgF or ABUW-2363 gene product was also identified, but its expression was invariant between the two growth conditions.

#### 2.3.3. Cell Envelope

Several enzymes involved in cell wall assembly, synthesis and stability were identified; however, their expression levels were found to be similar both in the absence and presence of IMP in the growth medium (Table 1). These enzymes include a ligase (DdlB), murein transglycosylases (MltB and Slt), a peptidotransferase (YkuD), an endopeptidase (MepM) and a peptidoglycan-associated lipoprotein (Pal) (Table 1). The lack of overexpression of enzymes involved in maintaining cell wall integrity suggests that carbapenemases as well as fine-tuning OMP permeability confer full protection from IMP in strain AB5075.

#### 2.3.4. Protein Synthesis

Three 50S ribosomal proteins were identified, two of them were shown to be essential for the growth of strain AB50745 in nutrient-rich medium (Table 1) [15]. Although these proteins are expected to be located in the cytoplasm, a previous study has reported the periplasmic space as their sub-cellular localization, corroborating the performance of SecretomeP in predicting the presence of an N-terminal export signal (Table 1) [11]. The expression of these ribosomal proteins was unaffected by the presence of IMP, in accordance with previously reported data [11].

#### 2.3.5. Transport and Binding Proteins

ATP-binding cassette (ABC) transporters are ubiquitous membrane proteins that couple the transport of diverse substrates across cellular membranes to the hydrolysis of ATP. They participate in many physiological and pathological processes. Herein, five periplasmic components of ABC transporters, the sulfur (Sbp*)*, the molybdate (ModA), the glutamate/aspartate (GltI), the phosphate (PstS) binding proteins involved in the transport systems for these nutrients, the phospholipid-binding protein MlaC and the cell surface protein DcaP-like were identified (Table 1) [44,45,46]. The MlaC protein is part of a multicomponent ABC transport system (*mlaFEDCB* operon and *mlaA*) that transport glycerophospholipids to the outer membrane to keep its integrity [47]. Accordingly, *mlaC* mutants displayed an increased susceptibility to antibiotics that enter through the outer membrane [47]. Further, PstS is a component of the *pstSACB* complex involved in the uptake of phosphate in several bacteria [22,48,49,50]. Conversely, structural studies of the DcaP-like protein showed that it was a trimeric outer membrane protein with a coiled-coil periplasmic domain that encompassed the first 60 amino acids in the mature form of the protein [51]. Therefore, identification of DcaP-like within the periplasmic fractions could be due to an outer membrane contamination or to its interaction with a periplasmic protein(s). Interestingly, a proteomic study highlighted that both PstS and DcaP-like proteins were involved in *A. baumannii* biofilm formation [52]. It was suggested that the upregulation of these proteins in biofilms was related to the increased demand of phosphate for exopolysaccharide production [52]. A BLAST search revealed that both PstS and the DcaP-like identified herein had identities of 99% with orthologues encoded by A1S_2448 (342/343) and A1S_2753 (433/434), respectively, in strain ATCC 17978. In view of these data, it is reasonable to suppose that both PstS and DcaP-like protein are also required during the exponential growth phase. None of the aforementioned proteins showed a significant differential expression in strain AB5075 between the two growth conditions.

#### 2.3.6. Fatty Acid and Phospholipid Metabolism

Within this functional category, it was found that the FabD, FabG and FabI (for fatty acid biosynthesis) proteins, encoded the malonyl-CoA:ACP transacylase, 3-oxoacyl-(acyl-carrier-protein) and enoyl- acyl-carrier-protein reductases (Table 1). These enzymes, belonging to the fatty acid biosynthesis pathway, are known to be located in the cytosol [53]. Therefore, their identification within periplasmic fractions represents a contamination event. Conversely, the periplasmic TesA protein is a multifunctional enzyme possessing activities of thioesterase, esterase, arylesterase, protease, and lysophospholipase belonging to the Gly–Asp–Ser–Leu (GDSL) family [54], previously found in *A. baumannii* [11]. As suggested for *P. aeruginosa*, TesA might regulate the composition of membrane lipids according to the environmental stimuli [55].

The ABUW_0040 belong to an uncharacterized subgroup of the steroidogenic acute regulatory protein (StAR)-related lipid transfer (START) domain family. A homologue with 100% identity was found in *K. pneumoniae* (UnirotKB entry A0A331R5K2). The STRING network predicted the interaction between ABUW_0040 gene product and AdeT based on their co-occurrence, although their function remains to be established (https://string-db.org/).

#### 2.3.7. Energy Metabolism

Four metabolism-related proteins were identified (Table 1); succinate-CoA ligase subunit alpha (SucD), glutaminase-asparaginase (AspQ), ribulose-phosphate 3-epimerase (Rpe) and inositol-1-monophosphatase (SuhB). In *E. coli*, the periplasmic type II L-asparaginase hydrolyzes Asn and, at a lower rate, also Gln for bacterial assimilation. Ribulose-phosphate 3-epimerase is involved in the pentose phosphate, succinyl-CoA ligase is an enzyme of the in the citric acid cycle, whereas the inositol-1-monophosphatase is an Mg^++^ dependent enzyme of the inositol phosphate metabolism, required to generate inositol for inositol lipids (https://www.genome.jp/kegg/). It was reported that Rpe has a β–barrel structure, which suggested an interaction with the bacterial inner membrane. The inositol-1-monophosphatase showed a 5-fold increase in strain AB5075 grown in the presence of IMP compared with LB grown cells (Figure 5). Although a defined role for SuhB has been missing, gene depletion showed pleiotropic effects in both *E. coli* and *Burkholderia cenocepacia* on cell envelope integrity*,* which affected protein secretion and stress response [56,57]. The pleiotropic phenotypes induced by the *suhB* deletion made SuhB an ideal target for the development of novel antimicrobials against MDR *B. cenocepacia* [57]. Aconitases are iron–sulfur proteins responsible for the reversible isomerization of citrate and isocitrate via the intermediate cis-aconitate in the TCA cycle [58]. Two genetically distinct aconitase proteins, AcnA and AcnB were found in several bacterial genomes, ABUW_3346 (*acnA*) and ABUW_1593 (*acnB*) in strain AB5075 [GenBank: CP008706.1]. Periplasmic AcnB is expressed during exponential growth, whereas AcnA is expressed in a stationary phase and under conditions of iron starvation and oxidative stress in *E. coli* [59]. The expression of AcnB was shown to be induced under iron-rich conditions in *A. baumannii* ATCC 19606 [60]. However, no differential expression was observed in energy metabolism enzymes following IMP exposure.

#### 2.3.8. Purines, Pyrimidines, Nucleosides, and Nucleotides

Purines and pyrimidines as well as their derivatives are essential for all living organisms, however, these nutrients are scarcely available exogenously. Herein, the carbamoyl-phosphate synthase small chain (CarA), N5-carboxyaminoimidazole ribonucleotide synthase (PurK) and the CTP synthase (PyrG) were identified. These enzymes are well conserved in all bacteria and some are essential for their biosynthetic pathways, as in the case of PyrG in *A. baumannii* [15]. To the best of the our knowledge, no subcellular localization has been described for them, with the exception of PyrG that was identified in the periplasmic fraction of *P. aeruginosa* [22]. No significant differences in the expression level was detected in the presence of IMP (Table 1).

#### 2.3.9. Cellular Processes, Amino Acid Biosynthesis, Biosynthesis of Cofactors, Prosthetic Groups, and Carriers and Biofilm Formation

The quinoprotein glucose dehydrogenase B (GdhB) is a periplasmic dimeric quinoprotein that requires pyrroloquinoline quinone as cofactor (PQQ). It catalyzes the oxidation of a broad range of aldose sugars to the corresponding lactones [61]. The cytoplasmic 2,3-dihydro-2,3-dihydroxybenzoate dehydrogenase enzyme (DhbA) was shown to be required for the biosynthesis of the acinetobactin precursor 2,3-dihydroxybenzoic acid (DHBA) in *A. baumannii* ATCC 19606, for iron acquisition [62]. The cytoplasmic UDP-N-acetyl-mannosamine dehydrogenase (MnaB) is an enzyme responsible for converting UDP-N-acetyl-mannosamine to UDP-*N*-acetyl-mannosaminuronic acid required for exopolysaccharides, such as cepacian in *Burkholdaria cepacia* or alginate in *P. aeruginosa* [63].

The triacylglycerol lipase (Lip1) is a member of the α/β-hydrolase fold family of enzymes, including structurally related proteins with diverse catalytic and non-catalytic functions. As in our case, catalytic members include hydrolases (acyltransferase, hydrolase, lipase) but lack experimental characterization of their enzymatic activity [64]. Interestingly, members of this family are assumed to be moonlighting proteins [65]. Therefore, Lip1 might exhibit different functions depending if it is retained within the periplasmic space or secreted extracellularly.

The 3-phosphoshikimate 1-carboxyvinyltransferase enzyme (AroA) is involved in the biosynthesis of chorimate, representing the precursor of all three aromatic amino acids, as well as cofactors (i.e., menaquinone, ubiquinone, salicylate and phenazine) (https://www.ncbi.nlm.nih.gov/Structure/cdd/cddsrv.cgi?uid = cd01556). PanC, the pantoate--beta-alanine ligase, is one of the four enzymes that lead to pantothenate biosynthesis from aspartate. Further, pantothenate is converted into to coenzyme A that is an essential molecule in the metabolism of living organisms, being a carrier of acetyl and acyl groups [66].

The *pgaABCD* operon encodes the proteins responsible for the formation and the translocation of the polysaccharide poly-β-(1-6)-*N*-acetylglucosamine (PNAG) across the outer membrane [67,68]. PNAG is the major component of biofilms that allows *A. baumannii*, as well as other microorganisms, to adhere to surfaces and give protection against antimicrobials, environmental stresses, and the host immune system [67]. It has been shown that N-deacetylase PgaB grants the periplasmic translocation of PNAG through its interaction with the outer-membrane polysaccharide secretin PgaA for substrate secretion [68]. There was no significant difference in protein expression between the two different growth conditions used in this study.

#### 2.3.10. Response to Oxidative Stress

Superoxide dismutases are key antioxidative enzymes widely distributed in organisms that protect the cell from harmful effects of reactive oxygen species (ROS). These metalloenzymes are classified based on the metal cofactor used. The major class uses manganese or iron ions as catalytic metal (SodA and SodB), while periplasmic superoxide dismutase uses copper and zinc ions as catalytic metal (SodC). Although generally considered to be a cytoplasmic enzyme [69], in this study, SodB was found in the periplasmic fractions (Table 1), as previously shown in several bacterial species [22,70,71,72]. Moreover, SodB was also identified within culture supernatants from *A. baumannii* [12]. The presence of SodB in the periplasmic space and extracellularly suggests that SodB is necessary to overcome exogenous sources of superoxide in addition to periplasmic SodC. Accordingly, a growing body of evidence supports the notion that several classes of antibiotics exert their bactericidal effects via the production of hydroxyl radicals [73]. It is reasonable to speculate that proteins involved in the defense against oxidative stress are upregulated or constitutively expressed in MDR strains, as AB5075. This conclusion is in line with recent findings demonstrating that SodB acts as an important antibiotic resistance factor in *A. baumannii* MDR strains [74]. Although different from SodB, a heat shock protein (Hs), encoded by ABUW_2868, was found to be upregulated in the presence of IMP in the growth medium (Table 1, Figure 5). Although poorly characterized, ABUW_2868 gene product was annotated as belonging to the HslJ-like superfamily. In *E. coli*, the HslJ protein was shown to be upregulated in response to antibiotic stress [75,76]. Therefore, it could be hypothesized that enhanced expression of the protein encoded by ABUW_2868 could be involved in the protection against the oxidative stress induced by IMP (Figure 5).

#### 2.3.11. Unknown Function

A large group of identified proteins consists of putative periplasmic proteins currently annotated as conserved hypothetical proteins, proteins containing domains of hypothetical proteins, or hypothetical proteins (Table 1). Among this category, three differentially expressed proteins, the putative lipoprotein ABUW_0459 gene product, the 7-cyano-7-deazaguanine synthase QueC (locus tag ABUW_1012) and the uncharacterized protein encoded by ABUW_2616 were found (Figure 5). QueC is a key enzyme in the biosynthesis of queuosine, a modified nucleoside incorporated in certain tRNAs to enhance translational fidelity [77]. It has been reported that *E. coli queC* mutants were viable but had impaired responses to nutritional stresses and virulence [78]. Therefore, one possible explanation for the upregulation of QueC in cells grown under IMP exposure might be that this protein is required to avoid adverse effects on translational fidelity under an antibiotic stress response.

### 2.4. Periplasmic Fractions from A. baumannii AB5075 Grown in the Presence of IMP Confer Greater Antioxidant Protection than Those Grown in LB

**Figure 6:**
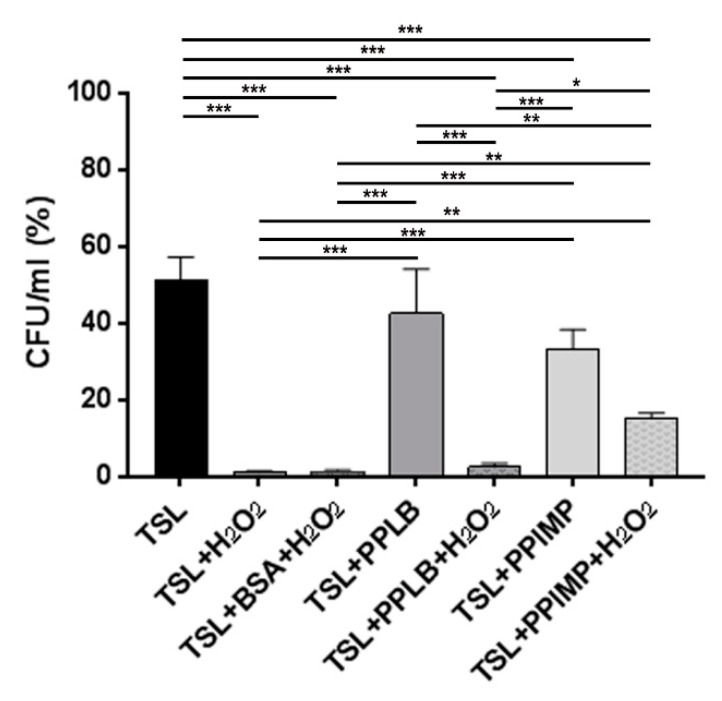
The survival rates of AB5075 cells incubated with periplasmic fractions extracted from *A. baumannii* cultures grown in the presence and absence of IMP and challenged with 2 mM of H_2_O_2_. Bacterial starting viability was controlled by re-suspending the inoculum used in this assay (1 × 10^6^/mL) in sodium chloride 0.9% (NaCl) which was set as 100% (not shown). Due to its composition, the periplasmic extraction buffer TSL (30 mM Tris-HCl–20% sucrose (pH 8.1), lysozyme 100 mg/mL in 0.1 M EDTA [pH 7.3]) was used as the control. The untreated and H_2_O_2-_treated bacteria resuspended in the periplasmic extraction buffer TSL in the presence of periplasmic extracts extracted from cells grown in LB (PPLB) or in LB supplemented with IMP (PPIMP). The untreated and H_2_O_2_-treated bacteria with or without the addition of 50 µg/mL of bovine serum albumin (BSA) to buffer hydroxyl radicals served as controls. Asterisks represent p values evaluated by one-way ANOVA; *** *p* < 0.001, ** *p* < 0.01, * *p* < 0.05.

To confirm that IMP induces the expression of periplasmic proteins with antioxidant capacity as HslJ*,* a survival rate test to hydrogen peroxide exposure was performed. Periplasmic fractions extracted from strain AB5075 grown in LB or LB supplemented with IMP were added to exponentially grown *A. baumannii* cells that were incubated with H_2_O_2_ (2 mM) before comparing survival rates measured as CFU/mL (see Material and Methods for details). Due to its composition, the treatment of AB5075 cells with periplasmic extraction buffer (TSL, Tris-HCl–sucrose-lysozyme) affected bacterial viability which dropped dramatically upon exposure to H_2_O_2_, as expected by its biocidal activity. The addition of an exogenous protein, as BSA, to buffer hydroxyl radicals by providing alternative targets did not increase significantly the survival rates of strain AB5075 (Figure 6). Interestingly, the addition of the periplasmic fraction extracted from AB5075 grown in LB supplemented with IMP before hydrogen peroxide showed no statistical difference in survival rates compared with those achieved by H_2_O_2_ unchallenged bacteria (TSL+PPIMP+H_2_O_2_ vs TSL+PPIMP, Figure 6). The same increase in bacterial survival was not observed in culture treated with the periplasmic fraction extracted from strain AB5075 grown in LB (Figure 6). Overall, these results indicate that strain AB5075 upregulates the expression of periplasmic proteins conferring protection against oxidative stress when cultured in the presence of IMP, as the overexpressed HslJ-like herein reported.

## 3. Conclusions

Carbapenems are powerful antibiotics for the treatment of MDR *A. baumannii* infections. However, the spread of carbapenem-resistant *A. baumannii* strains has narrowed dramatically the effective therapeutic options to eradicate these infections. The data presented herein show that resistance to IMP in strain AB5075 relays on increased expression of carbapenemases (i.e., OXA-23, GES-11 and MBL), as reported previously [11]. In addition, our data suggest that IMP, as other antibiotics, enhances its bactericidal activity through the production of hydroxyl radicals [73]. Although the periplasmic sub-proteomes of strain AB5075 grown in LB or in LB supplemented with IMP share a constitutive expression of the majority of the proteins, those involved in the defense against oxidative stress were upregulated, as demonstrated by the upregulation of HslJ-like (locus tag ABUW_2868) as well as the antioxidant activity assay. It is tempting to speculate that also the upregulation of the QueC may help translational fidelity under oxidative stress conditions.

In comparison to the cytoplasm, the periplasm is much more vulnerable to changes in the external environment, e.g., changes in pH, temperature, osmolarity and has a relatively higher oxidizing state. These physiological properties of the periplasmic space could account for the enhanced content of chaperones involved in protein folding and disulfide bond formation found in both growth conditions. Within the protein fate functional category, two proteins presenting the TPR motif were found to have opposite expression profiles in the presence and absence of IMP (YbgF and the protein encoded by ABUW_2363). The TPR motif is a protein-protein interaction module that facilitates specific interactions with a partner protein(s). Therefore, our hypothesis is that the switched expression of YbgF and ABUW_2363 gene products is an effective mechanism to adapt to changing environmental conditions by reorganizing the OMP content. Overall, it seems that MDR *A. baumannii* use different strategies to successfully cope with the stressful exposure to IMP. Undoubtedly, the comprehensive knowledge of IMP-upregulated proteins and their molecular functions could be crucial in designing new antibacterial molecules.

## 4. Materials and Methods

### 4.1. Bacterial Strain, Growth Conditions and Sub-Minimal Inhibitory Concentration (MIC) Determinations

The *A. baumannii* AB5075-UW strain (NR-49900) was obtained from the Biodefense and Emerging Infections Research Resources Repository for distribution by BEI Resources, the National Institute of Allergy and Infectious Diseases (NIAID), National Institutes of Health (NIH), while strain ATCC 17978 was a kind gift from P. Visca. The routine growth and plating were carried out in Luria-Bertani broth (LB) and 1.5% agar plates (Difco, Milan, Italy). The antibiotic susceptibility profiling was performed with the VITEK 2 system (bioMérieux, Florence, Italy). Imipenem (IMP) was purchased from Alfa Aesar (Italy). The MIC values were determined by MICROSCAN WalkAway (Siemens, Erlangen, Germany). To determine the appropriate imipenem sub-MIC values granting comparable growth curves, different concentrations of IMP (ranging from 1 to 8 µg/mL) were tested in a broth dilution assay. The growth kinetics were monitored by measuring the optical density (OD) at 600 nm and viable counts every 30 min (initial inoculum: 1 × 10^6^ colony-forming units per mL, CFU/mL) over a period of 8 h.

### 4.2. Periplasmic Protein Extraction

A 1:150 dilution of the overnight culture of strain AB5075 was used to inoculate 100 mL of LB (control) and LB supplemented with 6 µg/mL IMP. The bacteria were grown to a mid-exponential phase (OD_600_ ≈ 0.6) at 37 °C with vigorous shaking. To determine the most efficient method to obtain enriched fractions of periplasmic proteins from strain AB5075, three different procedures were tested. The chloroform-based method [17], the cold osmotic shock [18] and the method of Osborne and Silhavy [79] modified by Valenzuela and coll. [19]. To obtain a 2× periplasm fraction extracts, bacteria were re-suspended in half of the original volume of each protocol. For loading on gels, periplasmic fractions were concentrated by trichloroacetic acid (TCA) to a final concentration of 10% overnight, washed with cold acetone and re-suspended in 1 × Laemmli buffer [80]. The protein concentration was determined using the Lowry protein assay according to the manufacturer’s instructions (Bio-Rad, Milan, Italy).

### 4.3. Assay of Carbapenemase Activity

The three extraction methods were analyzed by a western blot with antiserum against SurA from *E. coli*. Unfortunately, no cross-reactivity of this antiserum was detected. Therefore, a plate assay was set up to test which method of extraction ensured the recovery of the higher carbapenemase activity in the periplasmic fraction. The carbapenem-susceptible strain *A. baumannii* ATCC 17978 was seeded as bacterial lawn on Mueller Hinton (MH) agar plates supplemented with IMP. Two microbiological disks, each containing 15 µL of 1× or 2× periplasmic fractions extracted with the three methods (20 and 40 µg/mL, respectively), were applied on the surface of the seeded plates. The disks containing 15 µL of the buffers used to extract the periplasm proteins were used as negative controls, whereas plates seeded with strain AB5075 served as positive controls. The plates were incubated at 37 °C for 24 h and photographed.

### 4.4. Assay of Antioxidant Activity

To determine if the periplasmic proteins possess any antioxidant activity, AB5075 cells were collected during the exponential growth phase, washed twice and re-suspended in 0.9% sodium chloride to a final concentration of 1 × 10^6^/mL. The periplasmic fractions, extracted from strain AB5075 grown in LB and LB supplemented with 6 µg/mL IMP, were added to the bacterial suspension, before adding H_2_O_2_ to a final concentration of 2 mM. The reaction was allowed to proceed for 10 min at 37 °C. Hence, the bacterial suspension was serially diluted and plated on LB agar plates to determine the CFU/mL after the treatment. The untreated and H_2_O_2_-treated bacteria re-suspended in the periplasmic extraction buffer (30 mM Tris-HCl–20% sucrose [pH 8.1], 100 mg/mL in 0.1 M EDTA [pH 7.3]), in absence of periplasmic extracts, with or without the addition of 50 µg/mL of bovine serum albumin (BSA) served as controls.

### 4.5. SDS-PAGE and In-Gel Digestion

Whole cell extracts (WCEs) and sub-fractions were re-suspended in Cracking Dye (2% SDS, 20% glycerol, 62.5 mM Tris-HCl pH 6.8, 0.05% bromophenol blue, and 5% β-mercaptoethanol), denatured for 10 min at 95 °C, and loaded onto a 12% SDS-PAGE. The resolved proteins were stained with Coomassie brilliant blue R-250 (Sigma, Milan, Italy). The gel lanes containing the periplasmic fractions were excised into 11 gel slices and subjected to in-gel digestion with trypsin (Promega, Milan, Italy) [81,82].

### 4.6. LC-MS/MS Analyses and Protein Identification

Tryptic peptides were subjected to nano-HPLC-ESI-MS/MS analysis performed on an Eksigent Ekspert Nano LC400 system (Sciex, Toronto, Canada) directly coupled to a TripleTOF 5600+ (Sciex) with a nanoESI source (NANOSpray III, Sciex, Toronto, Canada). The peptides were first trapped and desalted onto an Acclaim PepMap100 C18 column (5 µm particle size, 100 Å pore size, 100 µm i.d. × 2 cm, Thermo Fisher Scientific, Rome, Italy) for 5 min at 2 μL/min with solvent A (aqueous solution of 2% acetonitrile (ACN) and 0.1% formic acid (FA)) and then separated by reverse-phase chromatography on an Acclaim PepMap100 C18 column (3 μm particle size, 100 Å pore size, 75 μm i.d. × 25 cm, Thermo Fisher Scientific, Rome, Italy) at a flow rate of 300 nL/min and temperature of 40°C, by a two-step gradient of solvent B (ACN/water 2:98, *v/v*, final 0.1% FA) with chromatographic conditions as follows: linear gradient from 5 to 25% B in 60 min; from 25% to 60% B in 10 min and total LC-run of 95 min. Mass spectra were collected by the mass spectrometer operating in positive ion mode and in information-dependent acquisition (IDA) scan mode where each full mass spectrometric scan (mass range: 350–1250 *m*/*z*, acquired in 0.25 s) was followed by the isolation and fragmentation (MS/MS) of the 35 most abundant multiple-charged precursor ions via collision-induced dissociation (CID) with accumulation time set to 0.1 s from 230–1500 *m*/*z*. To avoid redundant sequencing of the most abundant peptides, the active exclusion was enabled for 15 s. The 11 raw data files for each lane were merged together in a single processing step by the ProteinPilot software (version 5.0.1, Sciex, Toronto, Canada). A databank search was performed versus the *A. baumannii* UniProtKB Protein Knowledgebase (release: 2017_11, 446,169 sequence entries), which employed both the embedded ion accounting Paragon algorithm and the Mascot server in house interface (version 2.3, Matrix Science Ltd., London, UK). Trypsin was set as proteolytic enzyme in both cases. Paragon search parameters were: iodoacetamide for cysteine alkylation; gel-based identification as special factor; biological modifications as identification focus; and false discovery rate (FDR) under 0.01 (i.e., the expected fraction of incorrect peptide spectrum match in the entire data set is less than 1%, calculated on a decoy database). All settings for mass tolerance were as the default for TripleTOF 5600+ mass spectrometer. To minimize false positive results, a strict cut-off for protein identification was applied, discarding protein matches with less than three peptide sequences with a 95% confidence. The Mascot search parameters were: one missed cleavage allowed; carbamidomethylation of cysteine as fixed modification; oxidation of methionine as variable modification; 10 ppm tolerance for precursor ions and 0.05 Da for product ions. The decoy search was enabled and the Mascot Percolator algorithm was used to minimize the FDR. The obtained emPAI values were used to perform a semi-quantitative evaluation of the protein relative abundance between biological experimental conditions [26].

### 4.7. Subcellular Localization Prediction

The functional annotation analysis of identified proteins was performed using the comprehensive bioinformatics tool UniProtKB. Subcellular localization, the presence of classical and non-classical signal peptides, putative transmembrane helices of identified proteins were predicted by PSORTb, version 2.0.4 (www.psort.org/psortb2/index.html), SignalP (www.cbs.dtu.dk/services/SignalP/), LipoP 1.0 (www.cbs.dtu.dk/services/LipoP/), PRED-TAT (www.compgen.org/tools/PRED-TAT/), SecretomeP 2.0 (www.cbs.dtu.dk/services/SecretomeP/), TOPCONS (http://topcons.cbr.su.se/pred/) and TMHMM (www.cbs.dtu.dk/services/TMHMM/). The identified proteins were classified into different categories based on the TIGR classification system (http://www.tigr.org) and on available literature of single proteins.

### 4.8. Statistical Analyses

Statistical differences were analyzed by one-way ANOVA tests using the Bonferroni post-hoc test and unpaired *t* test. The values of *p* < 0.05 were taken as being statistically significant. Statistical data analysis was performed using GraphPad Prism software (version 5.01, La Jolla, CA, USA).

## Figures and Tables

**Table 1:** Differentially expressed periplasmic proteins identified in *A. baumannii* AB5075 grown in LB or LB supplemented with IMP using a semi-quantitative approach.

UniProtKB Entry	Name	Locus Tag	Gene Name	PRED-TAT	SecretomeP 2.0	SignalP 4.1	LipoP 1.0	Functional Categories	*p*-Value of LB vs. IMP
				(SP, Signal Peptide; MLCS, Most Likely Cleavage Site; Rs, Reliability Score)	(Thresholds = 0.5)	(D-cutoff 0.57)	(SpI: Signal Peptidase I; SpIIsignal Peptidase II; CYT: Cytoplasmic)		(*t* test)
V5V8J3	AdeT RND type efflux pump	ABUW_0009	ABUW_0009	Sec SP MLCS: 1–22 [AQA-AS]. Rs: 0.998	0.796	0.879	SpI score = 23.7603	Antibiotic resistance	0.257
A0A059ZEN4	Polyketide cyclase/dehydrase	ABUW_0040	ABUW_0040	Sec SP MLCS: 1–21 [TGA-AS]. Rs: 0.989	0.873	0.740	SpI score = 12.0307	Fatty acid and phospholipid metabolism	0.775
A0A0D5YD86	PF09917 family protein	ABUW_0139	ABUW_0139	Sec SP MLCS: 1–19 [ANA-AD]. Rs: 0.999	0.942	0.908	SpI score = 22.236	Unknown function	0.586
A0A0D5YDD6	d-alanine-d-alanine ligase	ABUW_0148	*ddlB ^§^*	Sec SP MLCS: 1–22 [ERA-VS]. Rs: 0.975	0.132	0.154	CYT score = −0.200913	Cell envelope	0.241
A0A0B9W7U0	Putative porin	ABUW_0166	ABUW_0166	Sec SP MLCS: 1–22 [ANA-YQ]. Rs: 0.999	0.955	0.890	SpI score = 24.4957	Unknown function	0.660
A0A0D5YEL2	Inositol-1-monophosphatase	ABUW_0380	*suhB*	Sec SP MLCS: 1–28 [ARA-AQ]. Rs: 0.963	0.077	0.291	CYT score = −0.200913	Energy metabolism	0.034
V5V9K9	Putative phospholipid-binding protein MlaC	ABUW_0386	*mlaC*	Sec SP MLCS: 1–24 [AFA-AP]. Rs: 1.000	0.911	0.826	SpI score = 14.7115	Transport and binding proteins	0.655
A0A0D5YD51	Cytokinin riboside 5’-monophosphate phosphoribohydrolase	ABUW_0389	*yvdD_2*	Sec SP MLCS: 1–19 [IFQ-QI]. Rs: 0.914	0.076	0.212	SpI score = 0.389632	Unknown function	0.095
V5V9H7	50S ribosomal protein L2	ABUW_0409	*rplB ^§^*	No SP. Rs: 1.000	0.715	0.142	CYT score = −0.200913	Protein synthesis	0.556
A0A059ZGZ2	50S ribosomal protein L6	ABUW_0421	*rplF ^§^*	No SP. Rs: 0.906	0.921	0.185	CYT score = −0.200913	Protein synthesis	0.144
A0A0D5YET6	PF11306 family protein	ABUW_0459	ABUW_0459	Sec SP MLCS: 1–30 [ALA-MS]. Rs: 0.996	0.956	0.794	SpI score = 21.0497	Unknown function	0.035
A0A0D5YD61	Transglycosylase SLT domain protein	ABUW_0465	*slt_3*	Sec SP MLCS: 1–24 [SYA-AE]. Rs 0.998	0.821	0.820	SpI score = 16.2422	Cell envelope	0.801
Q9L4P2	OXA-23	ABUW_0563	*ari-1*	Sec SP MLCS: 1–20 [CTV-QH]–Rs: 0.934	0.125	0.522	SpII score = 7.79003	Antibiotic resistance	0.068
A0A090B0M2	Sel1 repeat protein	ABUW_0664	ABUW_0664	Sec SP MLCS: 1–19 [IFA-AD]. Rs: 0.998	0.948	0.924	SpI score = 19.7066	Protein fate	0.274
A0A0B9X9I7	DcaP-like protein	ABUW_0826	ABUW_0826	Sec SP MLCS: 1–29 [ANA-AT]. Rs: 1.000	0.957	0.760	SpI score = 13.4453	Transport and binding proteins	0.078
V5VBV8	Outer-membrane lipoprotein carrier protein	ABUW_0850	*lolA ^§^*	Sec SP MLCS: 1–28 [AFA-AP]. Rs: 1.000	0.952	0.927	SpI score = 25.1318	Protein fate	0.212
A0A0Q1DNT0	Succinate-CoA ligase [ADP-forming] subunit alpha	ABUW_0876	*sucD*	Sec SP MLCS: 1–28 [AQA-LD]. Rs: 0.943	0.302	0.140	CYT score = −0.200913	Energy metabolism	0.208
A0A0D5YF99	Carbamoyl-phosphate synthase small chain	ABUW_0894	*carA*	Sec SP MLCS: 1–22 [IGA-TG]. Rs: 0.986	0.269	0.204	SpI score = −0.0909364	Purines, pyrimidines, nucleosides, and nucleotides	0.098
A0A0D5YEE4	Metallo-beta-lactamase domain protein	ABUW_0920	*ytnP*	No SP. Rs: 0.939	0.932	0.101	CYT score = −0.200913	Antibiotic resistance	0.035
V5VAD5	Peptidoglycan-associated lipoprotein	ABUW_0992	*pal_1*	Sec SP MLCS: 1–29 [GDA-SG]. Rs: 0.990	0.912	0.583	SpII score = 22.7919	Cell envelope	0.374
V5VAE8	7-cyano-7-deazaguanine synthase	ABUW_1012	*queC*	Sec SP MLCS: 1–22 [AWA-QA]. Rs: 0.995	0.074	0.367	SpI score = 2.3942	Unknown function	0.037
V5VAT6	ABC transporter permease	ABUW_1021	*sbp_2*	Sec SP MLCS: 1–21 [SFA-AQ]. Rs: 0.996	0.848	0.833	SpI score = 13.9335	Transport and binding proteins	0.323
V5VAG2	Periplasmic serine endoprotease DegP-like	ABUW_1027	*mucD_1*	Sec SP MLCS: 1–24 [ANA-AV]. Rs: 0.999	0.888	0.660	SpI score = 8.19702	Protein fate	0.566
A0A0D5YFS0	Peptidase, M16 family	ABUW_1082	*ptrA*	Sec SP MLCS: 1–23 [SFA-QT]. Rs: 0.999	0.728	0.822	SpI score = 14.1822	Protein fate	0.099
V5VB00	PF04402 family protein	ABUW_1108	ABUW_1108	Sec SP MLCS: 1–21 [VFA-QD]. Rs: 1.000	0.885	0.809	SpI score = 14.5801	Unknown function	0.054
A0A0D8GKI2	Phosphate-binding protein PstS	ABUW_1115	*pstS_3*	Sec SP MLCS: 1–25 [ANA-AR]. Rs: 0.999	0.942	0.739	SpI score = 15.3657	Transport and binding proteins	0.750
V5VBD5	ErfK/YbiS/YcfS/YnhG family	ABUW_1189	*ykuD*	Sec SP MLCS: 1–21 [ALA-AS]. Rs: 0.999	0.950	0.916	SpI score = 18.7128	Cell envelope	0.942
D6NSM8	Beta-lactamase ADC7	ABUW_1194	*ampC*	Sec SP MLCS: 14–36 [IYA-GN]. Rs: 0.917	0.844	0.496	SpI score = 5.57966	Antibiotic resistance	0.206
A0A0D5YG57	Aminopeptidase	ABUW_1203	*pepN_2*	Sec SP MLCS: 24–49 [IFA-SN]. Rs: 0.996	0.618	0.528	SpI score = 1.53431	Protein fate	0.795
V5VAU8	Superoxide dismutase	ABUW_1216	*sodB*	No SP. Rs: 0.955	0.918	0.103	CYT score = −0.200913	Response to oxidative stress	0.270
V5VBL0	Lytic murein transglycosylase B	ABUW_1243	*mltB*	Sec SP MLCS: 1–28 [AQA-ND]. Rs: 0.999	0.826	0.783	SpI score = 15.767	Cell envelope	0.571
A0A090C137	3-phosphoshikimate 1-carboxyvinyltransferase	ABUW_1366	*aroA ^§§^*	Sec SP MLCS: 1–27 [IVA-EK]. Rs: 0.978	0.077	0.304	SpI score = 1.23597	Amino acid biosynthesis	0.194
V5VBJ4	Aconitate hydratase B	ABUW_1593	*acnB ^§^*	No SP. Rs: 0.989	0.386	0.091	CYT score = −0.200913	Fatty acid and phospholipid metabolism	0.099
A0A059ZHT5	Tetratricopeptide repeat protein	ABUW_1746	*ybgF_1*	Sec SP MLCS: 1–21 [LYA-NI]. Rs: 0.998	0.936	0.715	SpI score = 9.11005	Protein fate	0.019
A0A0D5YHX0	Quinoprotein glucose dehydrogenase B	ABUW_1762	*gdhB2*	Sec SP MLCS: 1–25 [AFA-DI]. Rs: 0.999	0.928	0.753	SpI score = 7.30243	Cellular processes	0.416
A0A086HXI4	CTP synthase	ABUW_1825	*pyrG ^§^*	Sec SP MLCS: 1–21 [ISA-AS]. Rs: 0.987	0.058	0.317	SpI score = 1.34202	Purines, pyrimidines, nucleosides, and nucleotides	0.137
A0A0D5YHL1	2,3-dihydro-2,3-dihydroxybenzoate dehydrogenase	ABUW_2076	*dhbA*	Sec SP MLCS: 1–19 [AIA-KQ]. Rs:0.971	0.103	0.243	SpI score = 4.55959	Cellular processes	0.374
A0A0D5YIT2	Molybdate ABC transporter substrate-binding protein	ABUW_2079	*modA*	Sec SP MLCS: 1–29 [AKA-ES]. Rs: 0.999	0.777	0.626	SpI score = 8.37936	Transport and binding proteins	0.512
A0A0D5YI49	Oligopeptidase A	ABUW_2224	*prlC_1*	Sec SP MLCS: 1–24 [ASA-EA]. Rs: 0,997	0.525	0.749	SpI score = 12.5788	Protein fate	0.989
A0A077GP18	Chaperone SurA	ABUW_2268	*surA ^§^*	Sec SP MLCS: 1–32 [SFA-QP]. Rs: 1.000	0.425	0.762	SpI score = 16.0755	Protein fate	0.160
V5XWY4	Polyisoprenoid-binding protein	ABUW_2293	ABUW_2293	Sec SP MLCS: 1–21 [TLA-AP]. Rs: 0.999	0.924	0.873	SpI score = 19.451	Unknown function	0.143
V5VDN7	Amino acid ABC transporter substrate-binding protein	ABUW_2333	*gltI*	Sec SP MLCS: 1–26 [IQA-AD]. Rs: 0.999	0.958	0.732	SpI score = 12.943	Transport and binding proteins	0.222
A0A077GKV3	Glutaminase-asparaginase	ABUW_2359	*aspQ*	Sec SP MLCS: 1–24 [LYA-KN]. Rs: 0.991	0.824	0.842	SpI score = 17.3321	Energy metabolism	0.559
V5VDR1	Tetratricopeptide repeat family protein	ABUW_2363	ABUW_2363	Sec SP MLCS: 1–24 [VYA-AR]. Rs: 0.988	0.127	0.427	SpI score = 7.46629	Protein fate	0.005
A0A0D5YK68	Uncharacterized protein	ABUW_2616	ABUW_2616 ^§§§^	Sec SP MLCS: 1–19 [SLV-DD]. Rs: 0.872	0.097	0.299	CYT score = −0.200913	Unknown function	0.024
A0A0D8GPY7	Uncharacterized protein	ABUW_2660	ABUW_2660	Sec SP MLCS: 1–20 [SYA-QS]. Rs: 0.996	0.842	0.749	SpI score = 13.0805	Unknown function	0.237
A0A0D5YJD8	Putative peptidase, M23/M37 family	ABUW_2738	*mepM_1*	Sec SP MLCS: 1–24 [AMA-EL]. Rs: 1.000	0.870	0.860	SpI score = 11.5496	Cell envelope	0.340
A0A0E1JMR4	Heat shock protein	ABUW_2868	ABUW_2868	Sec SP MLCS: 1–24 [SNT-QA]. Rs: 0.964	0.945	0.747	SpII score = 10.5977	Response to oxidative stress	0.048
A0A0D5YLD9	Acyl-CoA thioesterase	ABUW_2912	*tesA*	No SP. Rs: 0.439	0.944	0.233	CYT score = −0.200913	Fatty acid and phospholipid metabolism	0.179
A0A0D5YL61	Triacylglycerol lipase	ABUW_2914	*lip1*	Sec SP MLCS: 1–36 [AQA-AD]. Rs: 1.000	0.589	0.873	SpI score = 24.4375	Cellular processes	0.062
A0A0D5YHR1	Poly-beta-1,6-N-acetyl-D-glucosamine N-deacetylase PgaB	ABUW_2960	*pgaB*	Sec SP MLCS: 1–27 [ALA-NP]. Rs: 0.998	0.072	0.223	CYT score = −0.200913	Biofilm formation	0.481
V5VG70	LysM domain/BON superfamily protein	ABUW_3106	ABUW_3106	No SP. Rs: 0.830	0.918	0.108	CYT score = −0.200913	Unknown function	0.822
V5VHN7	3-oxoacyl-(Acyl-carrier-protein) reductase	ABUW_3108	*fabG_9 ^§^*	Sec SP MLCS: 1–22 [AIA-QQ]. Rs: 0.992	0.181	0.230	SpI score = 2.77001	Fatty acid and phospholipid metabolism	0.211
V5VFX0	Malonyl CoA-acyl carrier protein transacylase	ABUW_3109	*fabD_1 ^§§^*	No SP. Rs: 0.330	0.538	0.181	CYT score = −0.200913	Fatty acid and phospholipid metabolism	0.677
V5VGJ7	Ribulose-phosphate 3-epimerase	ABUW_3231	*rpe ^§^*	Sec SP MLCS: 1–20 [RLG-ED]. Rs: 0.731	0.076	0.207	CYT score = −0.200913	Energy metabolism	0.205
V5VGP5	Pantothenate synthetase	ABUW_3295	*panC ^§^*	Sec SP MLCS: 1–19 [ARA-AR]. Rs: 0.991	0.120	0.181	CYT score = −0.200913	Biosynthesis of cofactors, prosthetic groups, and carriers	0.085
A0A077GA87	Enoyl-[acyl-carrier-protein] reductase [NADH]	ABUW_3365	*fabI ^§^*	Sec SP MLCS: 1–29 [AQA-LH]. Rs: 0.968	0.0921	0.276	CYT score = −0.200913	Fatty acid and phospholipid metabolism	0.231
V5VH43	C-terminal processing peptidase family protein	ABUW_3385	*prc*	Sec SP MLCS: 1–28 [ARA-AT]. Rs: 0.998	0.682	0.592	SpI score = 7.74808	Protein fate	0.308
V5VH86	50S ribosomal protein L1	ABUW_3593	*rplA ^§^*	No SP. Rs: 0.218	0.946	0.168	CYT score = −0.200913	Protein synthesis	0.413
A0A0D5YME7	LysM domain/BON superfamily protein	ABUW_3722	ABUW_3722	Sec SP MLCS: 1–39 [VHA-TS]. Rs: 0.997	0.858	0.540	SpI score = 7.26442	Unknown function	0.124
A0A0F7YYU4	UDP-N-acetyl-mannosamine dehydrogenase	ABUW_3828	*mnaB*	Sec SP MLCS: 1–25 [TFA-AH]. Rs: 0.997	0.169	0.488	SpI score = 1.31476	Cellular processes	0.079
W8FNF9	Peptidyl-prolyl cis-trans isomerase	ABUW_3835	*fklB*	Sec SP MLCS: 1–19 [VFA-AA]. Rs: 0.991	0.135	0.904	SpI score = 19.8542	Protein fate	0.923
A0A0D5YNX0	Thiol:disulfide interchange protein	ABUW_3846	*dsbA*	Sec SP MLCS: 1–22 [AMA-AD]. Rs: 1.000	0.880	0.905	SpI score = 22.2592	Protein fate	0.204
A0A0D5YMJ1	N5-carboxyaminoimidazole ribonucleotide synthase	ABUW_3885	*purK*	Sec SP MLCS: 1–20 [AQA-AL]. Rs: 0.972	0.107	0.239	CYT score = −0.200913	Purines, pyrimidines, nucleosides, and nucleotides	0.323
C5HUY1	Beta-lactamase	ABUW_4052	*blaGES-11*	Sec SP MLCS: 1–18 [AYA-SE]. Rs: 0.997	0.092	0.604	SpI score = 3.88651	Antibiotic resistance	0.498

(*) Asterisks indicate those proteins in which the Tat 1.0 server recognized a potential Tat signal peptide but no Tat motif. (§) One section sign indicates essential genes, two non-essential genes, three the gene with no orthologue in strain ATCC 17978. Both TMHMM 2.0 and TOPCONS servers predicted no TMH and, therefore, was omitted.

## Supplementary Materials

Supplementary materials can be found at https://www.mdpi.com/1422-0067/20/14/3451/s1.
