# Supplementary material for: Insights into the Periplasmic Proteins of Acinetobacter baumannii AB5075 and the Impact of Imipenem Exposure: A Proteomic Approach
Source: Int J Mol Sci. 2019 Jul 13;20(14):3451. doi: 10.3390/ijms20143451 (PMC6679007; doi:10.3390/ijms20143451)
Supplement: Supplementary file 1 [file ijms-20-03451-s001.zip › Table S1.docx]

Table S1. Antibiotic susceptibility MICs of *A. baumannii* AB5075 performed by MICROSCAN WalkAway (Siemens).

| **Antibiotic** | **MIC (µg/ml)** | **EUCAST 2019** |
| --- | --- | --- |
| Ampicillin-sulbactam | >16 | N/A |
| Amikacin | >32 | R |
| Aztreonam | >16 | R |
| Ceftazidime | >32 | R |
| Cefotaxime | >64 | R |
| Chloramphenicol | ≤2 | NR |
| Ciprofloxacin | >2 | R |
| Cefepime | >32 | R |
| Doxycycline | <4 | R |
| Fosfomycin | 64 | R |
| Gentamicin | >8 | R |
| Imipenem | 8 | NR |
| Levofloxacin | >4 | R |
| Meropenem | 8 | R |
| Minocycline | ≤ 2 | N/A |
| Netilmicin | >8 | NR |
| Piperacillin-tazobactam | >64 | NR |
| Piperacillin | >64 | N/A |
| Trimethoprim-sulfamethoxazole | >4/76 | R |
| Tetracycline | ≤ 2 | R |
| Ticarcillin | >64 | N/A |
| Ticarcillin-clavulanic acid | >64 | N/A |
| Tobramycin | >8 | R |

MIC, minimal inhibitory concentration; EUCAST, European Committee on antimicrobial susceptibility testing; R, resistant, N/A, Not Applicable; NR: Not Reportable
